# In vitro or not in vitro: a short journey through a long history

**DOI:** 10.1186/s12302-018-0151-3

**Published:** 2018-06-26

**Authors:** Kristina Rehberger, Christian Kropf, Helmut Segner

**Affiliations:** Centre for Fish and Wildlife Health, Department of Infectious Diseases and Pathobiology, Vetsuisse Faculty, University of Bern, P O Box, 3001 Bern, Switzerland

**Keywords:** Cytotoxicity, Biotransformation, Bioaccumulation, Hazard profiling, In vivo, Risk assessment, Toxicity pathways, Prioritization, Screening, High-throughput

## Abstract

The aim of ecotoxicology is to study toxic effects on constituents of ecosystems, with the protection goal being populations and communities rather than individual organisms. In this ecosystem perspective, the use of in vitro methodologies measuring cellular and subcellular endpoints at a first glance appears to be odd. Nevertheless, more recently in vitro approaches gained momentum in ecotoxicology. In this article, we will discuss important application domains of in vitro methods in ecotoxicology. One area is the use of in vitro assays to replace, reduce, and refine (3R) in vivo tests. Research in this field has focused mainly on the use of in vitro cytotoxicity assays with fish cells as non-animal alternative to the in vivo lethality test with fish and on in vitro biotransformation assays as part of an alternative testing strategy for bioaccumulation testing with fish. Lessons learned from this research include the importance of a critical evaluation of the sensitivity, specificity and exposure conditions of in vitro assays, as well as the availability of appropriate in vitro-in vivo extrapolation models. In addition to this classical 3R application, other application domains of in vitro assays in ecotoxicology include the screening and prioritization of chemical hazards, the categorization of chemicals according to their modes of action and the provision of mechanistic information for the pathway-based prediction of adverse outcomes. The applications discussed in this essay may highlight the potential of in vitro technologies to enhance the environmental hazard assessment of single chemicals and complex mixtures at a reduced need of animal testing.

## Looking back…

The term “ecotoxicology” was coined, according to Truhaut [142], in June 1969 during a meeting of a committee of the International Council of Scientific Unions. The aim of ecotoxicology is to study the toxic effects on constituents of ecosystems, with the protection goal being populations and communities rather than individual organisms, as it is the case in human toxicology. In practice, however, ecotoxicology strongly relies on a classical toxicological testing approach with the emphasis on organism-level endpoints like mortality [28, 128, 137]. The reasoning behind this is that organismic endpoints are considered to bear ecological relevance as they may drive population growth rates. In this ecosystem perspective, the use of in vitro methodologies, measuring cellular and subcellular endpoints, appears to be odd. As a consequence, interest of ecotoxicologists in in vitro approaches was limited, and in vitro approaches had a niche existence in ecotoxicology for a long time [77]. The senior author well remembers the rejections of in vitro project proposals by environmental funding agencies due to “lack of ecotoxicological relevance”. Moreover, when attending his first conference on in vitro toxicology, the ESTIV (European Society of Toxicology In Vitro) in 1992 in De Haan, Belgium, he was the only ecotoxicologist among more than 200 human toxicologists, illustrating the rather limited interest of ecotoxicologists in this subject.

More recently, however, in vitro approaches gained momentum in ecotoxicology. This development was methodologically driven by the availability of new technologies [96] and conceptually driven by mainly three motivations. A first motivation was the utilization of in vitro techniques in basic studies to understand toxic mechanisms of environmental contaminants (e.g. [16, 30, 130]). A second motivation was the need for rapid screening of the toxic potential of a steadily growing number of chemicals and environmental samples. This promoted already in the late 1990s the application of so-called microscale assays such as the bacterial microtox assay, the pollen tube growth assay, cell-free preparations like the urease enzyme inhibition test or in vitro cell assays [20, 63, 138, 150]. The ability of the in vitro assays for high-throughput testing was also applied to support the establishment of Quantitative Structure–Activity Relationships (QSARs) [8, 44, 119]. Finally, in vitro assays were suggested as alternatives to in vivo ecotoxicity tests with vertebrates (e.g. [1, 9, 23, 31, 32, 120]). An important driving force behind the latter motivation was new chemical regulations such as REACH (Registration, Evaluation, Authorization and Restriction of Chemicals) in Europe, which brought along increased testing needs, implicating a boost in the use of experimental animals [74]. This situation further enhanced already existing ethical concerns on in vivo ecotoxicity tests (e.g. [1, 77]), and strongly encouraged the search for non-animal testing methodologies. We should keep in mind, however, that the trigger for this new interest of ecotoxicologists for in vitro approaches was largely motivated from ethical considerations and the question is whether they can contribute relevant and valid data for ecotoxicological hazard assessment, which is discussed controversially.

**Fig. 1:**
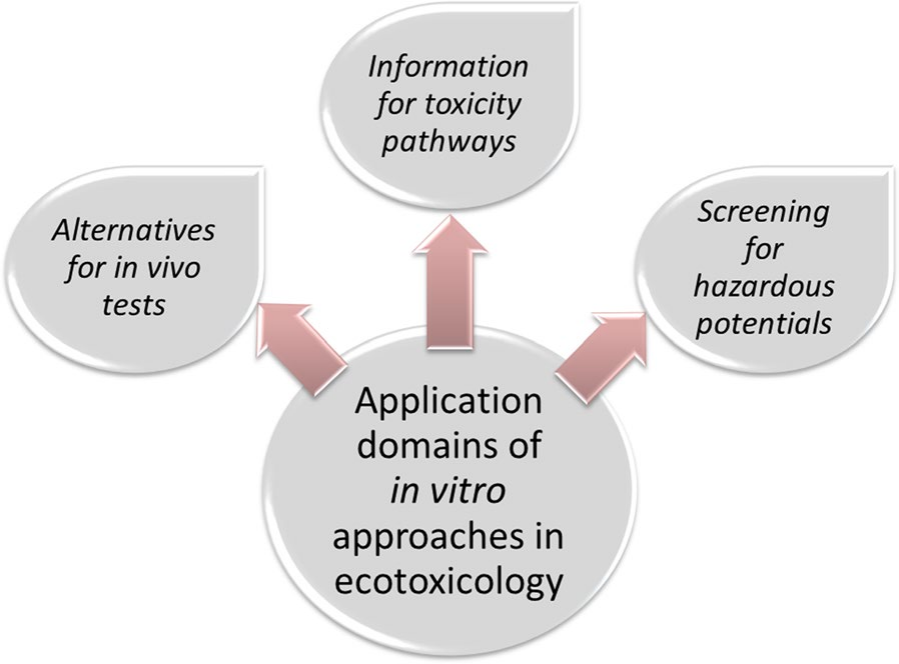
Examples for in vitro applications in ecotoxicology: In vitro approaches are widely used as alternative for in vivo tests in the sense of the 3Rs (replacement, reduction and refinement), to study toxic mechanisms at molecular and cellular levels and for screening of compounds or environmental samples for hazardous potentials

In the following, we will discuss two important application domains of in vitro methods in ecotoxicology, the use as alternatives to in vivo tests and the use for assessing hazardous potentials of chemicals (cf. Fig. 1), and we will critically discuss the scientific grounds for these applications. Like with every testing method, it is of key importance to clearly define for what purposes and what kind of questions they may be suitable, and what the possibilities and limitations are in extrapolating from in vitro data to the ecotoxicological target entities. In this context, we would like to emphasize that in our understanding, the term “in vitro” refers to cellular test and subcellular systems but excludes test systems like fish embryo tests.

## In vitro application domains in ecotoxicology: what kind of information can they provide?

### The 3R application domain of fish in vitro assays

Fish tests are the most frequently applied toxicity tests with vertebrates in regulatory ecotoxicology [31, 120]. For instance, the UK Annual National Statistics Report [143] provides the following numbers of non-mammalian species used in regulatory testing: 122’046 birds, no reptiles, 9951 amphibians, and 286’666 fishes. The fish numbers were higher than the number of rats (238’841) used in regulatory testing for human toxicology. Also, in an attempt to estimate the prospective animal test needs in human toxicology and ecotoxicology under REACH, the in vivo fish tests (long-term fish toxicity, fish bioaccumulation) were in the top ranks. Therefore, efforts to implement the 3R principle [117] in ecotoxicology were directed primarily towards in vivo fish tests.

The application of in vitro techniques for questions related to fish toxicology started as early as ecotoxicology emerged as scientific discipline. In 1968 Rachlin and Permutter [111] published a very first study using an in vitro assay with fish cells to assess metal toxicity to fish. From the middle of the 1990s, fish cell systems became a regularly used tool of ecotoxicological research. Pioneering work was done by Ellen Borenfreund and Harvey Babich who performed a series of studies using diverse fish cell lines to evaluate the cytotoxicity of a wide range of chemical classes [9]. The laboratory of Niels Bols succeeded in establishing diverse fish cell lines such as the RTL-W1 from rainbow trout (*Oncorhynchus mykiss*) liver or the RTgill-W1 from rainbow trout gills which can be used to detect specific toxicant responses such as the induction of cytochrome P450IA [12, 17, 39, 87, 88]. Such cell lines were particularly useful to derive toxicity equivalency factors for dioxin-like compounds and complex environmental samples [69, 153, 155]. In addition, fish cell lines were also used for purposes like the assessment of genotoxic (e.g. [83, 91, 100]), or immunotoxic activities of chemicals [16], or for the toxicity screening of complex environmental samples such as water effluents or sediment extracts (e.g.[2, 21, 34, 47, 63, 66]). The 1980s also saw the establishment of techniques for the isolation of primary fish cells, mainly hepatocytes [81, 95]. Since the liver plays a central role in toxicokinetic and toxicodynamic processes, isolated fish hepatocytes were broadly used for studies on toxic mechanisms, biomarker responses and xenobiotic biotransformation (for reviews, see [10, 24, 94, 98, 109, 124, 131]).

In the following, we will focus on two examples in establishing fish cell-based in vitro assays as alternatives to in vivo fish tests.

#### In vitro cytotoxicity assays with fish cells as alternative to the in vivo acute fish lethality test

**Fig. 2:**
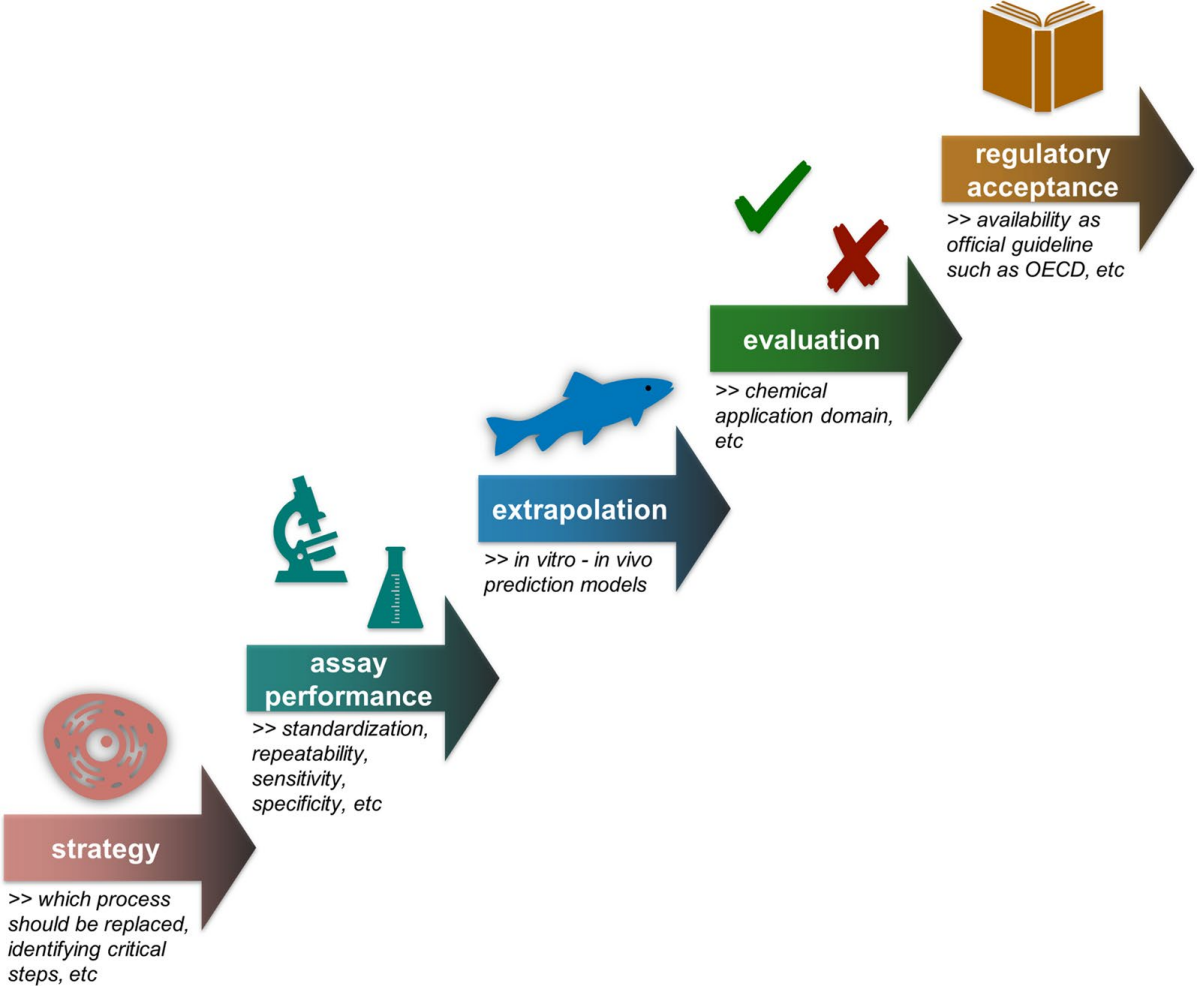
Crucial steps for developing and implementation of in vitro assays as an alternative for in vivo tests: First, steps and processes need to be defined in order to find out which target process could be replaced by in vitro assays. Next, assay performances including aspects such as test standardization have to be optimized, and it needs the development of appropriate in vitro–in vivo extrapolation models. Finally, the chemical application domain of the in vitro assays has to be identified. The last step, which is important for regulatory acceptance, is the implementation of an official Test Guideline, like the OECD Test Guidelines

Acute lethality tests with fish are frequently performed in regulatory hazard assessment as well as for wastewater effluent testing [106]. A standard procedure for the fish lethality test is described in the OECD Test Guideline 203 [107]. In vitro cytotoxicity assays with fish cells have been suggested as non-animal alternative to the in vivo lethality test with fish [2, 9, 15, 23, 31, 116, 120, 121]. The principal idea behind this suggestion is that the chemical concentration that causes cell death in vitro will also cause cell death in vivo, thereby leading to a lethal systemic failure of the organism. When aiming to establish an in vitro cytotoxicity assay to replace or reduce the in vivo lethality test, several key questions have to be answered (cf. Fig. 2): A first one relates to technical issues such as the selection of an appropriate infinite cell line or primary cell system, selection of suitable cell densities, the choice of the methods for measuring cell death/viability or the technical setup [17, 129, 147]. Next, it needs to be defined what type of information should be generated by the in vitro assay [80]—is it relative toxicity ranking or is it prediction of the in vivo LC50 (half maximal lethal concentration) values? If the latter applies, it will need a (quantitative) in vitro–in vivo extrapolation (IVIVE) model which takes into account the toxicokinetic differences between the in vitro and in vivo situation [14, 154].

The majority of in vitro cytotoxicity studies with fish cells relied—for practical reasons—on established cell lines rather than primary cells. Among the meanwhile up to 500 described fish cell lines [85], Cellosaurus https://web.expasy.org/cellosaurus, only a small percentage is publicly available, e.g. via cell banks like the ATCC (American Tissue Culture Collection) or the ECACC (European Collection of Authenticated Cell Cultures) and even a smaller number of cell lines has been used for cytotoxicity studies, including RTG-2, RTL-W1 and RTgill-W1 from rainbow trout (*Oncorhynchus mykiss*), PLHC-1 from the clearfin livebearer (*Poeciliopsis lucida*) or CHSE-14 from chinook salmon (*Oncorhynchus tshawytscha*; cf. [17, 31, 125]). The typical exposure period of the fish cells is 24 h and chemical toxicity is evaluated mainly as basal cytotoxicity. This term refers to cell death caused by toxicant-induced disruption of fundamental cellular features such as membrane integrity and mitochondrial energy generation [17, 54, 89, 147]. Other endpoints such as cell growth are rarely applied in cytotoxicity studies with fish cells, probably because the growth of fish cell lines is slow. Changes of membrane permeability can be assessed, for instance, by measuring the release of intracellular enzymes such as lactate dehydrogenase into the culture medium. One of the most frequently used methods in cytotoxicity studies with fish cells is the neutral red assay, introduced by Borenfreund and Puerner [18]. Neutral red is a weakly cationic dye that is retained only by viable cells. Altered metabolic activity of cells can be assessed by monitoring their ATP contents or their ability to reduce dyes such as MTT (3-(4,5,-dimethylthiazol-2-yl)-2,5-diphenyltetrazolium bromide) via enzymes of the mitochondrial respiratory chain to a spectrophotometrically measurable colored product [129].

The use of different cell lines and different methods for measuring cytotoxicity raises the question how much technical variations influence the outcome of in vitro cytotoxicity assays. Generally, there appears to be a good correlation between the different methods and cell lines. For instance, Saito et al. [118] found for the goldfish GFS cell line a high correlation of cytotoxicity results obtained with the lactate dehydrogenase cytotoxicity method and the MTT staining. Tan et al. [140] compared two methods for measuring cytotoxicity—MTT and the protein dye Coomassie Blue—and found high similarity between the two methods as well. Caminada et al. [29] compared the cytotoxicity of pharmaceuticals in the fish cell lines PLHC-1 and RTG-2, and found that for most compounds PLHC-1 was slightly more sensitive than RTG-2, but the differences were within an order of magnitude. In a study on nanoparticle cytotoxicity, Connolly et al. [40] found no significant differences between the results from fish cell lines and fish primary hepatocytes. The agreement of in vitro cytotoxicity data extends even from fish to mammalian cells, as reported by Castano and Gómez-Lechón [33] who observed a good correlation of IC50 values (half maximal inhibitory concentration of the chemical; *r* = 0.915) between fish and mammalian cell lines. These results are corroborated by the results of the MEIC study [36, 37], which compared growth and viability endpoints for 50 chemicals in more than 60 in vitro systems from animals and humans. They found a “remarkable similarity of all toxicity data” [36–38], irrespective of the choice of the endpoint or cell system. This similarity is because basal cytotoxicity, as it was measured in the assays of the MEIC study, arises from chemical interference with fundamental cellular features that are common to all cells and, therefore, should not show cell type specificity. However, exceptions from the generality of the basal cytotoxicity concept must not be overlooked. For instance, Segner [125], when performing a principal component analysis of the cytotoxicity of six chlorophenols to six fish cell lines, found that five cell lines grouped closely together whereas the PLHC-1 cell line displayed a dissimilar behaviour. Differences in toxicokinetic properties, i.e. cell-specific biotransformation, can contribute to different cytotoxicity outcomes between cell systems [36]. Also cell type-specific vulnerabilities of the membrane integrity or energy metabolism may lead to deviations from the expectations of the cytotoxicity concept [80, 133]. Last but not least, the choice of the cell system makes a difference if it comes to the study of specific modes of action [110].

The relative ranking of the toxicity of chemicals in vitro and in vivo generally correlates very well [31]. This was demonstrated in a number of studies: For instance, Bols et al. [15] found a significant correlation between the in vitro cytotoxicity of 12 aromatic hydrocarbons in a fish cell line, and their in vivo LC50 toxicity values. Likewise, several other studies reported high in vitro-in vivo correlations of relative toxicity ranking, too [7, 23, 32, 64, 116, 132]. Overall, the published data clearly indicate that cytotoxicity assays with fish cell lines are a valuable tool for the relative ranking of the in vivo fish toxicity of individual chemicals and effluents.

In contrast, the absolute sensitivity of in vitro cytotoxicity assays with fish cell lines appears to be clearly lower than the in vivo fish lethality test. Bols et al. [15] observed two to three orders of magnitude difference between the in vitro EC50 (half maximal effect concentration) and the in vivo LC50 values. Similarly, Kilemade and Quinn [80] as well as Segner [126] reported differences of two orders of magnitude between in vitro and in vivo. The sensitivity difference does not only apply for chemicals but also for complex environmental samples such as wastewater effluents. This was shown by a large-scale German study which compared the toxicity of more than 100 wastewater effluents in the acute lethality test with golden ide (*Leuciscus idus melanotus*) and in the in vitro cytotoxicity assay with the fibroblast-like R1 cell line from rainbow trout: Whereas the cytotoxicity assay classified 75% of the wastewater effluents to be of low fish toxicity (no more toxicity detectable at a wastewater dilution of 1:2), the fish lethality test classified only 41% of the samples into this category (unpublished data).

Absolute sensitivity, however, is of crucial importance in risk assessment since parameters like the Predicted No Effect Concentrations (PNEC) are derived from the toxic effect concentrations. If the in vitro effect concentrations are higher than the corresponding in vivo values, this will lead to non-protective PNEC values, and the use of in vitro cytotoxicity data instead of in vivo toxicity data would substantially underestimate the hazard of chemicals and effluents for the environment. The apparently low sensitivity of the in vitro assays is, therefore, a major stumbling block in their application for regulatory hazard assessment [31, 126]. The crucial point here is that the simple linear comparison of in vitro and in vivo effect concentrations does not take into account possible differences of chemical bioavailability in vitro and in vivo [14, 120]. The serum in the cell culture media as well as the plastic material of the culture plates for the cells can reduce chemical bioavailability to the cells by binding substantial fractions of the test chemicals [84]. As shown by Gülden et al. [67], converting the in vitro EC50 values into the free fraction EC50, i.e. the concentration that is not bound to serum, leads to a much better correspondence of in vitro and in vivo effect concentrations. This line of thinking was consequently further developed in the study of Tanneberger et al. [141]: Instead of nominal EC50 concentrations, these authors used the effective EC50 concentrations, i.e. the chemical fraction that is freely available to the cells and found a very good agreement between the effective EC50 values of 35 organic chemicals in the RTgill-W1 cytotoxicity assay and the in vivo LC50 values of the test compounds. For up to 73% of the test chemicals, the difference between the in vitro and the in vivo data was less than fivefold, thus, much lower than what is observed in comparisons on the basis of nominal concentrations (see above). Appropriate toxicokinetic prediction models can further improve the estimation of in vivo toxic concentrations from in vitro cytotoxicity data. A number of such models have been developed over the last decade in human toxicology (e.g. [13, 154]), but corresponding approaches are now emerging in fish toxicology as well [25, 136].

In conclusion, many lessons were learned by ecotoxicologists from the long journey in developing in vitro cytotoxicity assays with fish cells, including the critical evaluation of which in vivo effect parameters should be replaced by which in vitro assay, the setting up of technical standards for in vitro assays, and the learnings on the factors influencing sensitivity. The knowledge obtained from this research was beneficial to the development of other in vitro alternatives in ecotoxicology like the in vitro biotransformation assays.

#### In vitro biotransformation assays as part of an alternative strategy to in vivo bioaccumulation testing with fish

Information on the bioaccumulation potential of chemicals is a key parameter required in regulatory risk assessment. For the aquatic environment, the standard method to assess chemical bioaccumulation is the OECD Test Guideline 305 [108] which measures the bioconcentration factor (BCF) in fish. The BCF expresses the steady-state concentration of a chemical in the fish versus the concentration in the surrounding water. The drawback of the in vivo BCF determination with fish is that the test is lengthy, costly, and requires a high number of animals (> 100 fishes per test). With the implementation of new chemical regulations such as REACH, there is a growing need for bioaccumulation data, what would implicate a major increase in test animal usage [48]. This situation stimulated the search for alternatives to the in vivo BCF test with fish.

As a first step in developing alternatives to the in vivo bioaccumulation assessment with fish, the Health and Environmental Sciences Institute (HESI) organized a series of workshops which started in the 2000s, and discussed options of generating BCF data for fish with less use of animals [102, 149]. In silico hydrophobicity models can well predict the lipophilicity-based accumulation of xenobiotics in fish as a result of a passive partitioning process between water and fish [5, 48]. A disadvantage of the in silico models is that currently they cannot account for the influence of biotransformation on xenobiotic bioaccumulation and, therefore, they overestimate the BCF values for compounds which are metabolized. The HESI workshops identified the influence of xenobiotic biotransformation on the BCF to represent a major sources of uncertainty in the bioaccumulation assessment in fish. In vitro biotransformation assays may provide the required information to correct lipophilicity predicted BCF values for the influence of biotransformation [102, 149].

The use of in vitro assays to study xenobiotic biotransformation in fish dates back to the 1980s. Mainly preparations from the liver such as the S9 fraction, the microsomal fraction or isolated hepatocytes were employed (e.g. [10, 43, 65, 131]) but also established fish cell lines (e.g. [135]). The majority of these studies were interested in identifying the xenobiotic metabolites rather than predicting in vivo bioaccumulation. Nevertheless, these assays are principally suitable to generate data on xenobiotic biotransformation rates in the liver of fish. These data may then be used to correct the predictions of the in silico models and/or may be used for the direct prediction of the in vivo biotransformation rates of the chemicals.

Once biotransformation assays using in vitro liver preparations from fish had been identified as candidates for an alternative BCF assessment strategy, the next step was to adapt and standardize the available in vitro assay protocols (cf. Fig. 2). The assay systems included fresh suspensions of isolated liver cells [51, 62, 73] and liver S9 fractions [78]. The model species used in this research was rainbow trout. The technical optimization of the in vitro assays included also the development of cryopreservation techniques for the reliable provision of the biological material [93]. In the next step, the intra- and inter-laboratory reproducibility of the in vitro biotransformation assays had to be evaluated. The findings show an overall good intra- and inter-laboratory repeatability of the data [61]. The final step in the methodological development was to draft an OECD Test Guideline for the in vitro assays and to test the repeatability of the guideline in an international ring test [103]. With April 2018, the test guidelines for the in vitro biotransformation assays with rainbow trout hepatocytes and S9 fractions have been accepted by the OECD.

A lesson learned from the development of the cytotoxicity assays with fish cells was the importance of appropriate IVIVE models. Thus, this aspect was considered right from the beginning in the development of in vitro biotransformation assays. The prediction models enable the extrapolation of in vitro biotransformation rate values into in vivo biotransformation rates of the fish, allowing the calculation of a predicted BCF value [42, 102, 104]. In addition to physiological data with relevance for the toxicokinetics, the extrapolation models also consider the in vitro/in vivo bioavailability of the xenobiotics which is a critical factor influencing the outcome of the prediction [6, 25, 59, 82, 105]—another important lesson learned from the studies on the cytotoxicity assays (see above). For the chemicals tested to date for in vitro biotransformation, a good agreement between in vitro predicted and empirically measured BCF values was observed [61, 73, 86]. What is still lacking is that the in vitro assays are tested with a broader array of chemicals in order to elucidate their chemical application domains.

Taken together, the biotransformation assays provide a good example of the targeted development of an in vitro alternative to an in vivo test (cf. Fig. 2), starting from a critical literature review through pioneering laboratory scale studies over assay standardization and repeatability evaluation, to a technology that is mature for regulatory acceptance. It is important to highlight that the in vitro biotransformation assays are not intended as a full replacement of the in vivo OECD 305 test [108], but as part of a tiered weight-of evidence approach to bioaccumulation assessment [90]. In a first tier, in silico methods would identify those compounds which are unlikely to show significant bioaccumulation based on their physicochemical properties; this step sorts out already a substantial amount of chemicals resulting in a reduction of testing needs [101]. In a next step, the in vitro biotransformation assays can deliver information whether the compound is likely to be metabolized by fish and at which rates. The in vitro values are then extrapolated by appropriate models to predict in vivo BCF values. In this scenario, the utilization of the in vivo fish bioconcentration test would only be applied in doubtful cases when the bioaccumulative potential of the test chemical cannot be unequivocally classified by the in silico and in vitro methods.

### The “hazard profiling” application domain of in vitro assays

The previous chapters discussed the use of in vitro assays with fish cells or subcellular fractions to replace, reduce and refine in vivo *fish* tests that measure apical toxic effects of chemical exposure, for instance, lethality. Historically, apical endpoints have been and will continue to be key endpoints in the regulatory ecotoxicological risk assessment. In our opinion, in vitro assays at the current state of scientific knowledge are ready to reduce animal usage for the assessment of apical endpoints, without compromising reliability and soundness of risk assessment.

In addition to the 3R use of in vitro tests, new application fields for in vitro assays in ecotoxicology have been emerging more recently. This is related to a paradigm change in ecotoxicology that is driven by mainly two issues: One issue is that ecotoxicology is confronted with a steadily growing number of chemicals and environmental samples to be tested. This can no longer be mastered by conventional in vivo testing approaches but necessitates the use of rapid and cost-effective technologies to screen, rank and prioritize the huge number of test agents and to alert for potential hazards [4, 49, 50, 52, 72]. Here, in vitro systems are the method of choice for the rapid profiling of the hazardous properties of chemicals and environmental samples. The second issue is the growing awareness that ecotoxicology has to move beyond an empirical, descriptive compound-by-compound testing of chemicals but has to give increasing emphasis on the understanding of the modes of action and toxicity pathways in order to be able to categorize chemicals and to predict adverse outcomes [22, 53, 68, 92, 113, 128]. A key trigger to this new pathway-oriented perspective was probably the endocrine disruption case that attracted attention to the pathway linkages between subtle molecular and physiological changes and the ecological effects [3, 127, 139]. In vitro assays can provide information on the initial steps of the toxicity pathways and this information may then be used for the predictive assessment of adverse outcomes [3, 35, 146].

Batteries of in vitro assays can screen for a wide range of specific hazardous potentials and reactivity of test agents such as endocrine activity or dioxin-like activity, both on single chemicals as well as on complex environmental mixtures [52, 56, 152]. Furthermore, in vitro assays offer a number of technical advantages: They are rapid to perform, of small scale, rather simple to conduct, cost-effective, and at least partly suitable for (automated) high-throughput testing. The results lead to the prioritization of chemicals for further testing and they provide guidance how to structure the subsequent testing. From early attempts of using in vitro assay batteries (e.g. [114, 134, 151]), this field has experienced a rapid growth, also because of the availability of new technologies such as reporter assays or genomic methodologies (e.g. [58, 70, 112, 144]). A recent example is provided by the US ToxCast programme, which profiles concentration-dependent responses of chemical inventories across a battery of in vitro assays including cell-free systems, cell lines and primary cells to detect chemical interference with specific molecular pathways and functions as well as with cellular stress responses and cytotoxicity (e.g. [50, 79]).

The hazard profiling information provided by in vitro assays is of value for the mode of action (MOA) classification of chemicals. The MOA refers to the set of molecular, cellular, physiological or organismic responses upon exposure to a toxicant (cf. [27, 60, 148]). In ecotoxicology, MOA assignment of chemicals is often done based on structural rules and physicochemical descriptors [55, 122]. The structure-based approaches for MOA classification, although being highly useful, also have inherent limitations. Therefore, it has been suggested to enhance the structure-based approaches by biological response profiles, with in vitro assays being the appropriate technological tool to provide the response data [46, 75, 115, 123].

Importantly, the in vitro-based approaches for hazard profiling are suitable not only for individual chemicals but also for the bioanalytical assessment of complex environmental samples [19, 41, 56, 71]. Panels of in vitro assays are increasingly used as effect-based tools to monitor the chemical quality status of the environment. The results of in vitro profiling can prioritize sampling locations, identify hot spots of contamination or diagnose the joint toxicity potential arising from the mixture of all active chemicals and metabolites being present in the environmental sample [19, 27, 45, 99]. In combination with effect-directed analysis, in vitro tools can also help to establish cause-effect relationships [26]. Overall, the in vitro tools provide a valuable complement to the targeted chemical analysis, which is still the most commonly used tool for environmental monitoring. In contrast to targeted chemical analyses, the effect-based tools respond also to the activity of the unknown, non-analysed compounds and their mixture effects [17, 153]. A possible drawback in the use of effect-based tools is, however, the difficulty to define what level of bioassay response is acceptable with respect to the quality status of the environment. Nevertheless, there are numerous discussions ongoing addressing this question (e.g. [57]), and solutions are underway.

The information provided by in vitro assay on the molecular and cellular actions of toxic chemicals is of value not only for hazard profiling and MOA categorization of chemicals but may also be integrated into toxicity pathway concepts for the prediction of adverse outcomes of chemical exposure. This perspective was largely stimulated by the report of the National Research Council [97] on “Toxicity Testing in the 21st century”. Contrary to classical (eco-)toxicology which starts hazard assessment at the level of adverse whole animal effects as measured in conventional toxicity tests, the concept of “Toxicology Testing in the 21st century” understands toxicity as the outcome of chemical-induced alterations in molecular pathways and cellular networks of the organism. Based on this, the report suggested a new testing paradigm which relies strongly on “toxicity pathways”, i.e. the paths leading from molecular and cellular responses to adverse health outcomes. In such a concept, in vitro assays can provide valuable information on the early events in the toxicity pathways. The “adverse outcome pathways” (AOPs, [3]) provide a conceptual framework for the linkages between molecular initiating events, as they may be measured in vitro, and toxic effects at the organism or population levels. A critical aspect in this concept is the transition between the various biological levels. To link between different biological responses within an AOP, certain conditions have to be fulfilled such as concentration–response relationships, essentiality, and biological plausibility, as formalized in the Bradford–Hill criteria for AOP [11]. To integrate in vitro data into an AOP framework, it needs—again—toxicokinetic information and models to translate the in vitro effect concentrations into in vivo threshold values. It will be a major challenge for future research to establish tools and concepts to quantitatively translate the data generated by in vitro assays into toxicity pathways and AOPs.

## Looking forward

The title of this short essay asks “in vitro or not in vitro?”. Hopefully, we could provide convincing arguments that the answer is clearly positive for the use of in vitro assays in ecotoxicology and that this is supported not only from ethical but also from scientific grounds. In fact, in vitro assays can enhance the currently used approaches in ecotoxicological hazard assessment. While in vivo tests such as the fish acute lethality test bear little ecological relevance [28], a battery of in vitro assays generating a toxicity profile of a chemical or environmental sample and informing on the initial steps of adverse outcomes can provide in-depth information on ecological functions at risk. In combination with genomic technologies, in vitro assays offer ample opportunities for a more informed hazard assessment, both in chemical testing and in the monitoring of the environment. As such, they are an important tool in moving descriptive approaches to a more systematic and predictive assessment of the environmental risk of chemicals and complex mixtures.

Challenges for the use of in vitro assays in ecotoxicology, however, remain. One major shortcoming is to translate effect concentrations determined in vitro into effect concentrations of the intact organism. Here, it probably needs different approaches for environmental samples and for the regulatory testing of chemicals. In the first case, concepts like the effect-based trigger values appear very promising to overcome the “watershed” between in vitro and in vivo. For chemical testing, physiologically based toxicokinetic models are urgently needed. In human toxicology, much emphasis has been placed on this essential link from in vitro to in vivo, but ecotoxicology still lags behind—what is at least partly due to the fact that the available physiological information, which is needed to parameterize the models, is very limited for most ecotoxicologically relevant species. Another bottleneck is the development of in vitro alternatives to complex in vivo endpoints such as chronic toxicity. While classical 3R methods may be sufficient to tackle endpoints like acute lethality or bioaccumulation, the complex endpoints will need new technologies and concepts. Here, the integration of in vitro data in toxicity pathways might be particularly valuable. Finally, the regulatory acceptance of in vitro methods in ecotoxicological risk assessment remains a bottleneck, too. In vitro assays might be applied to replace an existing animal test, or as a weight-of-evidence element in an “Intelligent Testing Strategy” (ITS; e.g. [90]). For the regulatory use of in vitro assay, it usually needs a validated OECD Test Guideline. An obstacle in validation of in vitro assays for hazard assessment in ecotoxicology can be the high variability of the in vivo test data, which exists despite the use of Good Laboratory Practice and standardized Test Guidelines [76]. Until very recently there was no OECD validated fish in vitro assay available, which had changed with the recent acceptance of the OECD Test Guidelines for biotransformation assays with fish hepatocytes and S9 fractions. The question is now how widely these assays will actually be used and come to regulatory acceptance.

Overall, starting from rather shaky grounds some 20–30 years ago, in vitro assays have made their way in ecotoxicology and there are good reasons to believe that the full potential of in vitro approaches in ecotoxicology has been not exploited yet.

## Abbreviations

AOPs: adverse outcome pathways
BCF: bioconcentration factor
EC50: half maximal effect concentration
ESTIV: European Society of Toxicology In Vitro
HESI: Health and Environmental Sciences Institute
IC50: half maximal inhibitory concentration
IVIVE: in vitro–in vivo extrapolation
LC50: half maximal lethal concentration
MOA: mode of action
OECD: Organization for Economic Co-operation and Development
PNEC: Predicted No Effect Concentrations
QSARs: Quantitative Structure–Activity-Relationships
REACH: Registration, Evaluation, Authorization and Restriction of Chemicals
